# DNA motif alignment by evolving a population of Markov chains

**DOI:** 10.1186/1471-2105-10-S1-S13

**Published:** 2009-01-30

**Authors:** Chengpeng Bi

**Affiliations:** 1 Bioinformatics and Intelligent Computing Lab, Division of Clinical Pharmacology, Children's Mercy Hospitals, Kansas City, Missouri, USA; 2 Schools of Medicine, and Computing and Engineering, University of Missouri, Kansas City, Missouri, USA

## Abstract

**Background:**

Deciphering *cis*-regulatory elements or *de novo *motif-finding in genomes still remains elusive although much algorithmic effort has been expended. The Markov chain Monte Carlo (MCMC) method such as Gibbs motif samplers has been widely employed to solve the *de novo *motif-finding problem through sequence local alignment. Nonetheless, the MCMC-based motif samplers still suffer from local maxima like EM.

Therefore, as a prerequisite for finding good local alignments, these motif algorithms are often independently run a multitude of times, but without information exchange between different chains. Hence it would be worth a new algorithm design enabling such information exchange.

**Results:**

This paper presents a novel motif-finding algorithm by evolving a population of Markov chains with information exchange (PMC), each of which is initialized as a random alignment and run by the Metropolis-Hastings sampler (MHS). It is progressively updated through a series of local alignments stochastically sampled. Explicitly, the PMC motif algorithm performs stochastic sampling as specified by a population-based proposal distribution rather than individual ones, and adaptively evolves the population as a whole towards a global maximum. The alignment information exchange is accomplished by taking advantage of the pooled motif site distributions. A distinct method for running multiple independent Markov chains (IMC) without information exchange, or dubbed as the IMC motif algorithm, is also devised to compare with its PMC counterpart.

**Conclusion:**

Experimental studies demonstrate that the performance could be improved if pooled information were used to run a population of motif samplers. The new PMC algorithm was able to improve the convergence and outperformed other popular algorithms tested using simulated and biological motif sequences.

## Background

Discovering *cis*-regulatory elements or DNA motifs in genomic sequences is fundamental to build genetic networks and important to understand gene regulation in biological and pathological processes [1]. Although much algorithmic effort has been expended, it still remains challenging (see recent reviews in [2,3]). Multiple sequence local alignment coupling with position weight matrix (PWM) updating, or PWM-based technique for short, has been widely used to solve the *de novo *motif discovery problem [2,3]. This problem is of combinatorial optimization, and it has proven NP-complete [4]. A motif model or PWM can be uniquely defined by a local alignment, and the PWM updating is applied to progressively calibrate the alignments until some specified criterion is met. In the following text, the terms local alignment and motif discovery are often interchangeable. Basically, the PWM updating technique consists of two broad approaches to approximating the local alignment solutions, deterministic and stochastic motif-finding algorithms. The deterministic local alignment algorithm developed by Lawrence and Reiley in 1990 [5] is rooted on the Expectation Maximization (EM) method [6]. EM has been widely applied in various scientific computing since its introduction due to its simplicity and efficiency [7]. The EM motif algorithm has spawned a plethora of its variations (see review in [8] and references therein), for example, a popular motif algorithms called MEME is an enhanced EM version [9].

The stochastic local alignment method is the second approach in the PWM updating. It has its origin in Markov chain Monte Carlo (MCMC) methods [10] pioneered by Metropolis et al. in 1953 [11] and generalized later on by Hastings [12]. The earliest MCMC-based motif discovery algorithm is called Gibbs motif sampler developed in 1993 [13], and later many other MCMC-based motif samplers have been investigated such as BioProspector [14]. Moreover, the EM algorithm relates to the MCMC algorithms in the sense that it can be viewed as a forerunner of the Gibbs sampler in its data augmentation version [10,15,16], also as seen in a recent comparative study on these two approaches to detecting protein domains [17]. The MCMC algorithms have some appealing features better than EM, for example, they can escape from the local optima suffered by the EM algorithms. Most importantly, MCMC can be used as a general framework for solving a wide range of complex problems where EM often fails, because for these problems computing the expectation or maximization steps as required in EM often become infeasible. In particular, MCMC provides a framework for drawing samples from a complicated target distribution that cannot be sampled with simpler deterministic methods.

In practical sequence alignment, the MCMC sampling algorithms may also suffer from local maxima like EM [3]. To remedy this limitation, a MCMC motif algorithm is often run a multitude of times each starting from different points, that is, there is a population of Markov chains running in parallel. This simple strategy is strongly recommended and should be able to efficiently explore the probability landscape with multiple modes [3]. The population-based Monte Carlo methods serve two purposes, that is, to improve or diagnose convergence [10,18] and escape from local maxima [3]. It can be classified into two categories: (1) running multiple independent Markov chains (IMC) without any information exchange between chains, and (2) enabling information exchange among a population of Markov chains (PMC). It is straightforward to implement IMC for any MCMC-based samplers. In real world, IMC is often encouraged in running MCMC motif samplers. However, as up to now PMC is rarely concerned with in sequence local alignment [3]. In literature, PMC exists in a diversity of forms. For example, parallel tempering (PT) evolves R Markov chains each attaching a different temperature [19]. Chains in PT exchanges information by swapping two states that are generated by mutation, and accepted by a modified Metropolis-Hasting rule. Note that PT is the population-based simulated annealing algorithm and it only exchanges information pairwise. The real PMC algorithm should be able to exchange information at the population level, for example, the evolutionary Monte Carlo (EMC) [20] and population MCMC [21]. This paper presents the PMC motif-finding algorithm, a novel local alignment method. It evolves a population of Markov chains (PMC) with information exchange, each chain being updated according to the population-based or pooled proposal distributions and Metropolis-Hasting rule. Experimental studies demonstrate that the new algorithm was able to improve the convergence as well as evolve some better local alignments while compared to IMC and other motif algorithms tested.

## Results

### Metropolis-Hastings sampler for local alignment

A Metropolis-Hastings motif sampler (MHS) was first devised to carry out multiple local alignment. MHS run a single Markov chain initialized from a random alignment seed, which is the so-called initial alignment (**A**^(0) ^with *t *= 0 step). A motif model or PWM matrix Θ^(*t*) ^at step *t *can be derived from such a sequence alignment (**A**^(*t*)^). Then a Monte Carlo simulation is processed as follows: first scanning all sequences by the motif model built at step *t*. The scanning process simply calculates the probabilities of each potential motif sites on the input sequences using equation (1). Second, new samples are drawn from each sequence according to proposed distributions built from the sites scanned in the step *t*. Third, a energy function as defined in equation (3) is calculated, and the Metropolis-Hastings acceptance rate defined in equation (6) is applied to decide whether or not the new samples (i.e. new alignment) are passed to the next iteration. Finally, the above procedure is iterated for a number of iterations or cycles (*C*). A parallel MHS (i.e IMC) run the MHS motif sampler a multitude of times (*R*) independently without any information exchange, whereas the population-based MHS (i.e. PMC) run the same MHS sampler *R *times with information exchange enabled among all chains.

#### Scanning function

The information weight function originally proposed by Kullback and Leibler in 1951 [22] is used to approximate the probability of a potential site (*a*_*i*_) on sequence *i *(*S*_*i*_) given the current motif model Θ^(*t*)^,

(1) $$p(a_{i}|A^{\left(t\right)},\Theta^{\left(t\right)})\propto \sum_{j=a_{i}}^{a^{\prime }}\sum_{k=A}^{T}\log{\left(\frac{\theta_{j^{\prime }k}^{\left(t\right)}}{\theta_{0k}}\right)}^{\delta (S_{ij},k)}$$

where *a' *= *a*_*i *_+ *w *- 1, *j' = j *- *a*_*i *_+ 1, and *δ *is the indicator function. The information weight function simply indicates how similar a motif nucleotide probability (${\vec{\theta }}_{j}$) is relative to the background (${\vec{\theta }}_{0}$). Therefore, a site gets a larger scanning probability if its motif sequence is more different than the background. The scanning function can be derived in the EM motif algorithms [5,8] or Gibbs motif samplers [13,14,17].

#### Scoring function

Given a set of unaligned sequences (**S**), each local alignment can be treated as a configuration (*v*) in the whole local alignment space. The potential energy of a configuration (i.e. a local alignment **A**^(*t*)^) is defined by,

(2) *H*(*v*) = *H*(Θ^(*t*)^, **A**^(*t*)^, **S**),

where **A **is the missing data (i.e. the motif sites), **S **is the observed data and Θ is a parameter matrix (or a motif model) to be estimated. Note that the potential energy has its real biological meaning, for example, it may indicate the protein-DNA binding affinity [23,24] or energy of a protein three-dimensional configuration [10]. Assuming the Boltzmann distribution, one can compute the probability of such a configuration (*p*(*v*)). Although the energy function *H*(·) can be defined in any forms of interests, here a simple local alignment log-likelihood function [8,9] is used, which is given as,

(3) $$H(A^{\left(t\right)})\equiv Q(\Theta^{\left(t\right)})=|A^{\left(t\right)}|\sum_{j=0}^{w}\sum_{k=A}^{T}\tau_{t}(\theta_{jk}),$$

where |**A**| is the aligned motif sites, and $\tau_{t}(\theta_{jk})=-\theta_{jk}^{\left(t\right)}\log(1.0/\theta_{jk}^{\left(t\right)})$ is a negative entropy function. The energy function described as above and its variants have been widely adopted in maximum likelihood [5,8,9,17] or maximum a posterior based local alignment algorithms [10,13,14].

#### MHS sampling

The probability of a local alignment follows the Boltzmann distribution as,

(4) *p*(**A|S**) = *Z*^-1 ^exp{-*λH*(**A**)},

where *λ *= 1/*k*_*B*_*T*, and *Z *is a normalization constant. Given the current local alignment (**A**^(*t*)^, Θ^(*t*)^), a new local alignment (**A**^(*n*)^) is proposed according to the following proposal distribution,

(5) $$P(A^{\left(n\right)}|A^{\left(t\right)})=\prod_{i=1}^{N}p(a_{i}|A^{\left(t\right)},\Theta^{\left(t\right)}).$$

As noted, the proposal distribution assumes each sequence is independently sampled. The proposed alignment is either accepted or rejected according to the Metropolis-Hastings rule, and its acceptance rate (*α*_*H*_) is defined as,

(6) $$\alpha_{H}=\min\left\{1,\exp(\lambda \Delta H)\frac{P(A^{\left(n\right)}|A^{\left(t\right)})}{P(A^{\left(t\right)}|A^{\left(n\right)})}\right\},$$

where Δ*H *= *H*(**A**^(*n*)^) - *H*(**A**^(*t*)^). The transition probability from alignment **A**^(*t*) ^to **A**^(*n*) ^is thus expressed as follows: *T*(**A**^(*n*)^|**A**^(*t*)^) = *P*(**A**^(*n*)^|**A**^(*t*)^)*α*_*H*_(**A**^(*n*)^|**A**^(*t*)^). The MHS algorithm keeps updating the current best alignment and associated maximum energy along the sampling process or Markov chain. The output is the final best alignment (A*) and its corresponding maximum energy (*H*_*max*_) as well as the associated best motif model (Θ*). It should be pointed out that the energy maximization is equivalent to the minimization, that is, the optimizations max{*H*} and min{-*H*} are the same.

The IMC version of the MHS motif algorithm can be easily implemented in parallel. Let *R *independent Markov chains each starting from a different initial alignment, and then the IMC algorithm run *R *chains separately. Thus, one can send each run (i.e. a single MHS sampler) to a different compute node for execution. A set of the best alignments collected from all samplers in nodes can be sorted out and output the top best alignments as the potential solutions (note that the top-10 solutions are output as the default).

### The PMC motif-finding algorithm

Running multiple independent Markov chains (i.e. IMC) was originally designed to evaluate convergence [15], and later was found of its efficiency in improving convergence [18]. The goal of multiple local alignment is to uncover the target motifs hidden in a set of unaligned sequences as fast as possible. This requires that a MCMC motif-finding algorithm be able to efficiently explore the alignment space and in the same time locate as many near-optimal motifs as possible. In addition, to be practical, a Markov chain should be quickly converged since biological sequence alignment is a quite large-scale problem. As mentioned before, IMC technique is used in motif-finding on regular basis. However, it does not allow information exchange between chains. The idea of the PMC motif-finding algorithm is simply proposing the sampling distribution based on the current population of alignments, rather than on each individual. Since the transition matrix is based on the current population, the transition matrix of a single chain may be not stationary, but it evolves as a whole population which is stationary. It is hoped that such population-based information exchange would be able to improve the motif-finding performance and convergence as well. The PMC motif algorithm first initializes a population of R independent alignments (**A**^(*r*0)^: *r *= 1, ⋯, *R*). Each individual alignment at step *t *(**A**^(*rt*)^) can be uniquely mapped to a PWM model Θ^(*rt*)^, which can be used to scan sequences the same as in a single MHS motif sampler described as above. Now the proposal distribution is based on the current population of alignments **A**^(*rt*)^. After scanning step, each individual often generates different distributions of site probabilities, *p*($a_{i}^{r}$|**A**^(*rt*)^): *r *= 1, ⋯, *R*, here *p*($a_{i}^{r}$|·) is the site probability on location (*a*_*i*_) of sequence *i *in the *r*-th Markov chain. To summarize the current population of site probabilities on each sequence location (*a*_*i*_), its expectation $\bar{p}$(*a*_*i*_|*R*, *t*) and variance *σ *(*a*_*i*_|*R*, *t*) can be estimated as follows,

(7) $$\bar{p}(a_{i}|R,t)=E[p(a_{i})]=\sum_{r=1}^{R}p(a_{i}^{r}|A^{\left(rt\right)})/R;$$

(8) $$\sigma^{2}(a_{i}|R,t)=\sum_{r=1}^{R}\frac{{\left[p(a_{i}^{r}|A^{\left(rt\right)})-\bar{p}(a_{i}|R,t)\right]}^{2}}{R-1}.$$

One would argue that a larger variance (*σ*) may lead to a slow convergence. Thus the population-based proposal distribution is defined as,

(9) $$\bar{P}(A^{\left(rn\right)}|A^{\left(rt\right)})=\prod_{i=1}^{N}\bar{p}(a_{i}|R,t),$$

where *r *= 1,⋯, *R*. Each individual alignment so proposed is either accepted or rejected according to the Metropolis-Hastings rule, and the population-based acceptance rate (*α*_*P*_) is defined as follows,

(10) $$\alpha_{P}=\min\left\{1,e^{\lambda \Delta H^{r}}\frac{\bar{P}(A^{\left(rn\right)}|A^{\left(rt\right)})}{\bar{P}(A^{\left(rt\right)}|A^{\left(rn\right)})}\right\},$$

where Δ*H*^*r *^= *H*(**A**^(*rn*)^) - *H*(**A**^(*rt*)^). The *α*_*P *_rule is independently applied to all chains in the population.

This would ensure that each chain evolves independently, but adaptively progresses as a member in the population towards a unified direction.

### Comparison of sampling trajectories

**Figure 1:**
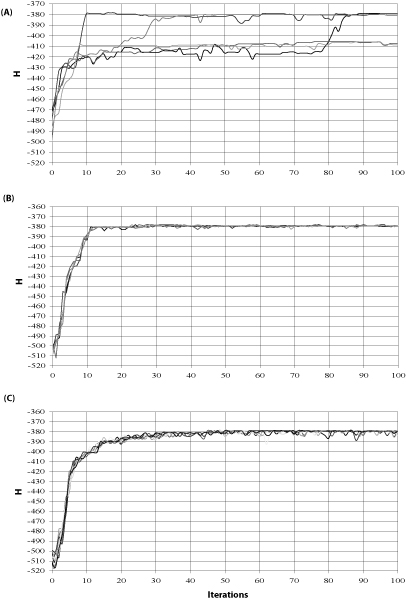
**Trajectories of MC chains**. The trajectories were plotted using the CRP binding data. Both IMC and PMC run the same MHS algorithm *r *times, but with different strategies as described in the text. (A) IMC with *r *= 5, (B) PMC, *r *= 5, (C) PMC, *r *= 10. Each arrow points to the alignment with the highest likelihood *H*_*max*_.

The trajectories were plotted using the original CRP binding data set. The IMC version run the MHS algorithm 5 times (i.e. *R *= 5), each being independent. The PMC algorithm was run in a population of either 5 or 10 individual chains, and the population evolved together. The sampling trajectories were plotted in Figure 1A–1C. Five chains were largely differing in the IMC case, that is, some converged quickly whereas others were very slow (Figure 1A). Note that these chains started from different initializations and ended up with distinct trajectories or equilibrium points. For the population-based motif algorithm (i.e. the PMC version), all chains (i.e. *R *= 5 or 10) quickly converged to the same global equilibrium level, as displayed in Figure 1B and 1C. This nicely illustrated that PMC improved the convergence in each chains. Moreover, the PMC alignment with *R *= 5 reached its maximum *H*'s around iteration 24, whereas the PMC alignment with *R *= 10 achieved its maxima around iteration 45. This reveals that a larger population may slow down the convergence possibly due to a bigger variance (*σ*^2^). Besides, more chains in the population may generate more dominant motif sites that compete for the equilibrium points. As a result, it should spend more time in burn-in phase before reaching a global equilibrium as more chains are added to the population. However, a larger population could explore more space. It is very hard to determine an optimal size, as it may be data-dependent.

On the other hand, the IMC version took the longest to come up with the best alignment at iteration 93 in 3 out of 5 chains. In addition, the IMC chains ended up with different results (Figure 1A), because some initial seeds result in good alignments with high energy (*H*), but others in bad alignments with low *H*. This nicely demonstrates that a prerequisite for achieving good alignment solutions would be running multiple times of a MCMC-based sampler, which provides more opportunities of being able to search more space and thus escape from local maxima, as already suggested in the literature [3,17]. As noted in Figure 1B–1C, all PMC chains quickly converged to the same equilibrium, which produced a global optimal solution rather than a set of different solutions exhibited in IMC (Figure 1A).

**Figure 2:**
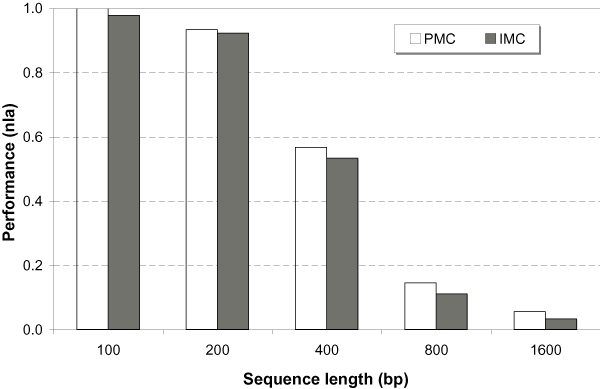
**Performance comparison**. The CRP binding motifs were planted in simulated background sequences with different lengths. Both IMC and PMC showed the same trend, that is, their performances go down as the sequence becomes longer.

Figure 2 summarizes the algorithm performance in different length groups of simulated CRP binding data sets. Both IMC and PMC versions successfully detected the planted motifs when *L *≤ 400 bp. PMC performed slightly better than IMC in each case. Overall, both performance dropped off as the background sequence becomes longer. They failed when the sequence length *L *≥ 800 bp. This is because a long random background sequence provides high chance of generating decoy signals that are highly conserved. To systematically test this kind of subtle motif discovery and further show the capability of the PMC motif-finding algorithm, the following section tested the well-formulated subtle motif problem.

### Subtle motif discovery

The planted (*l*, *d*)-motif problem is a typical subtle motif-finding problem first considered by Sagot [25], and later was rigorously formulated by Pevzner and Sze [26]. Here the motif width is denoted as *l *instead of *w *in order to be consistent with the original problem definition. Since this problem came to exist, it has been challenging every motif-finding algorithms without exception [27]. It is commonly thought that position weight matrix (PWM)-based motif algorithms such as the EM motif-finders [5,9] and Gibbs motif samplers [13,14] are unable to solve the problem [26,27]. This section revisited the planted motif problem using the IMC and PMC algorithms developed in the paper, and compared with two other algorithms, Weeder [28] and Projection [27], which were specifically designed for tackling the planted problem. Table 1 summarized the performance comparison of the five motif discovery algorithms tested in the 10 planted (*l*, *d*)-motif cases. One can divide the 10 cases into two categories: the 5 easy cases, i.e. (11,2), (13,3), (15,4), (17,5) and (19,6), and the 5 tough cases, i.e. (10,2), (12,3), (14,4), (16,5) and (18,6). The easy cases are those planted motifs with slightly high conservation, whereas the tough cases with relatively low conservation. Overall, the PMC algorithm outperformed other algorithms with the grand average *nla *= 0.65. Assuming that a case is successfully detected if more than half planted sites are uncovered (*nla *≥ 0.5). The PMC algorithm successfully detected almost all planted motifs with *nla *ranging from 0.55 to 0.93, and it failed in two very tough cases, (16,5) and (18,6). Among these detected cases, PMC predicted better than others in most cases (see the bolded numbers in Table 1) except for two cases, that is, Projection had the best *nla *= 0.84 in (17,5) and IMC with the best with *nla *= 0.96 in (11,2). Nonetheless, PMC still performed quite well in the two cases with *nla *= 0.93 in (11,2) and *nla *= 0.76 in (17,5). Projection is the second best predictor with average performance of *nla *= 0.53. Projection effectively located the 4 easy cases, (11,2), (13,3), (17,5) and (19,6) with *nla *= 0.95, 0.85, 0.84, 0.64, respectively. PMC achieved higher precision than Projection in the tough (10,2) case with *nla *= 0.73 vs. 0.53, as well as in the (19,6) case with *nla *= 0.75 vs. 0.64 as manifested in Table 1. Projection did not succeed in uncovering the remaining 4 tough cases: (12,3), (14,4), (16,5) and (18,6). Notably, previous report on Projection demonstrated its excellent performance in all planted cases [27], whereas this report showed discrepancies on it using the new unbiased motif data with the carefully chosen background sequences (see Methods). The difference may imply that Projection's performance is likely heavily dependent on the composition of the planted motifs used and their background sequences as well. Notice that although Projection adopted the random projection technique to pre-select a set of initial aligning seeds, it runs the EM algorithm as its final step that still suffers from local optimum. Taken together, results show that Projection did significantly improve the EM algorithm's performance (compare the average *nla*: 0.53 vs. 0.41), nonetheless, it still suffers from local maxima especially in subtle cases. The experiments allowed Projection to know the exact *d*'s in each cases, that is not required in other algorithms (and thus it may be not fair to other algorithms compared).

**Table 1:** Comparison using (*l, d*)-motifs. Note that here EM is DEM [8]. The number in bold corresponds to the best predictor in that case row.

	Algorithms				
(*l*, *d*)	WEE	PRO	EM	IMC	PMC
10,2	0.46	0.53	0.32	0.42	**0.73**

11,2	0.74	0.95	0.47	**0.96**	0.93

12,3	0.27	0.30	0.29	0.22	**0.59**

13,3	0.44	**0.85**	0.43	0.72	**0.85**

14,4	0.29	0.25	0.31	0.21	**0.55**

15,4	0.27	0.67	0.40	0.50	**0.75**

16,5	0.20	0.16	0.32	0.13	0.28

17,5	0.23	**0.84**	0.59	0.39	0.76

18,6	0.20	0.12	0.34	0.14	0.32

19,6	0.20	0.64	0.58	0.26	**0.75**

ave	0.33	0.53	0.41	0.40	**0.65**

The IMC and EM motif-finders performed nearly equal on average (i.e. 0.40 vs. 0.41). These poor results achieved are in tune with previous reports [26,27], that is, the EM and MCMC-based motif algorithms had difficulties in solving the planted motif problem. IMC only correctly predicted three easy cases: (11,2), (13,3) and (15,4) with average *nla *= 0.96, 0.72 and 0.50, respectively, whereas EM barely uncovered two easy cases with long motif widths: (17,5) and (19,6) with *nla *= 0.59 and 0.58, respectively. Notice that the PMC algorithm is significantly better than its independent counterpart IMC in almost all the simulated cases. It is evident that the PMC motif algorithm is superior to IMC, see the 25% increment on average performance (i.e. 0.65 vs. 0.40). However, in finding highly conserved motifs, IMC and PMC may perform approximately the same, as will be shown in the JASPAR benchmarks.

Weeder only detected the (11,2) case and failed the remaining cases with average performance of *nla *= 0.33. Previous studies showed that Weeder was one of the best motif predictors in mammalian promoter regions [29], it may imply that a highly heterogeneous word frequency distribution made a major contribution to its success in real biological cases, whereas the word frequencies in the simulated backgrounds were nearly uniformly distributed, that may render the motif-finding much more difficulty to Weeder. In addition, Weeder may be preferred in non-oops motif discovery, because its strategy is that selecting as many significant words as possible and put all of them in the candidate list. In difficult situations, while other algorithms totally fail, Weeder may still have the chance to find some meaningful signals. However, such winning strategy becomes the weakness in *oops *motif discovery, because the more motifs reported, the higher false positives incurred. Currently, there is no way to force Weeder run as an *oops *model.

### Testing JASPAR benchmark

In order to test the algorithm performance using the JASPAR data sets, one needs to generate simulated promoter sequences since the original JASPAR sequences are too short (about 25 bp on average). The simulated eukaryotic promoter sequences were generated by zero-order Markov model and they were composed of 45% GC content, which conforms to the ENCODE promoter regions [1]. Each JASPAR binding site was implanted into one simulated promoter sequence of length 500 bp. Note that the longer the sequence, the harder to locate the target as demonstrated in Figure 2. The 500-bp long sequence is approximately the same size as in a ChIP-chip data set, and should be a moderate size to challenge each algorithms tested. The Weeder and Projection programs were run the same as in the planted motif cases. For Projection, when *l *= 9 bp, *d *is still set to 2 mismatches, and when *l *= 20 – 22 bp, *d *= 7 – 8 mismatches as an extension to the original problem. Here the EM motif algorithm used is the popular MEME [9] run as its defaults.

Table 2 summarized the results of the five motif-finding algorithms. Compared to the planted motif problem, all algorithms tested using the JASPAR data carried out excellent jobs of finding almost all the experimentally verified motifs, the overall performance ranging from *nla *= 0.58 to 0.84. The PMC algorithm is still the best predictor with the average *nla *= 0.84, and IMC is very close to it with accuracy at *nla *= 0.82. MEME and Projection performed equally well with the average accuracy at *nla *= 0.75. Like in the planted motif cases, Weeder is the poorest predictor with average *nla *= 0.58 in the JASPAR data sets. In fact, Weeder predicted about 67% sites correctly, however, it also predicted more non-sites, and thus its average performance was decreased a bit. Notice that in the *w *= 9 case, both PMC and IMC predicted correctly whereas others failed.

**Table 2:** Comparison using JASPAR. Note that here EM is MEME [9].

	Algorithms				
*w*	WEE	EM	PRO	IMC	PMC
9	0.38	0.39	0.43	0.53	0.53

10	0.55	0.65	0.63	0.65	0.77

11	0.48	0.73	0.74	0.80	0.80

12	0.73	0.79	0.84	0.88	0.90

13	0.21	0.75	0.77	0.82	0.85

14	0.65	0.82	0.82	0.91	0.87

15	0.90	0.90	0.90	0.97	0.97

16	0.53	0.79	0.73	0.85	0.95

20	0.82	0.90	0.89	0.94	0.94

ave	0.58	0.75	0.75	0.82	0.84

The above experimental results illustrated the ability of the PMC algorithm to predict the binding sites not only in the simulated subtle motif data, but also in the experimentally verified data. PMC did outperform its independent counterpart IMC. In other words, information exchange between multiple Markov chains as implemented in PMC improved the convergence and the motif prediction as well. If a population of MHS motif samplers evolves towards a common target function, it would be expected that the performance might be improved, because pooled information is used to inform each individual proposal distribution. Suppose an individual chain from one sampler starts from a bad point and it definitely results in a poor solution while evolving independently. Now, if the population information is used to define its proposal distribution, that would render the poor chain much better with the benefit of being a member, as illustrated in Figure 1.

## Discussion

This paper presents a novel PMC motif-finding algorithm by evolving a population of Markov chains. The PMC motif algorithm exchanges local alignment information between individual chains by applying a pooled proposal distribution to all chains, and thus each individual chain can adaptively evolve towards a population-level equilibrium or global target function. Experimental studies demonstrate that the new PMC algorithm was able to improve the convergence and evolve better alignment solutions while compared to its multiple independent Markov chains method (IMC) and other algorithms. Further investigation into the population MCMC methods may be as follows: (1) disturbance added to the PMC chains may assist in exploring the alignment space, for example, one can incorporate genetic operators such as crossover and mutation into the PMC procedure; (2) fine-tuning sampling parameters might be informative to boost the accuracy of subtle motif discovery.

On the other hand, genetic algorithms [30], a class of adaptive global search methods modeled after biological systems, have been recently tried to overcome the limitations inherent in EM [31] as well as in MCMC [32]. It can be expected that computational intelligence techniques, which include genetic algorithms, neural networks and swarm intelligence, are likely to play important roles in sequence motif alignment.

## Methods

### Multiple sequence local alignment

The motif discovery problem is simply defined as: finding some recurrent short sequence patterns or motifs that are likely embedded in a given set of biological related sequences (**S**), for example, upstream promoter regions of co-regulated genes or enriched binding sequences in ChIP-chip experiments among others. Multiple local alignment is the most widely used method to locate over-represented sites in a given sequence set. The aligned over-represented sites are then used to build a frequency matrix that depicts a conserved domain or motif. Let **S **= {*S*_1_, ..., *S*_*i*_, ..., *S*_*N*_} denote the sequence data set. Let *L*_*i *_be the length of the sequence *i *(*S*_*i*_) and *S*_*ij *_denote a residue symbol taking on a value in *K*, for instance, *K *= {*A*, *C*, *G*, *T*} is an alphabet of DNA sequences. If only one motif per sequence (i.e. *oops *model) is assumed, there are *N *motifs in total for *N *sequences. A zero or one motif per sequence (i.e. *zoops*) model is also frequently used. Nonetheless, both *oops *and *zoops *models assume that sequence data come from a two-component multinomial mixture model: (1) the background model, assuming that residues at non-motif positions follow an independent and identical multinomial distribution (${\vec{\theta }}_{0}$); and (2) the *w*-mer motif model (or *w*-motif), assuming that residues within the motif are independent but not identical, in other words, residues at different motif positions come from different multinomial distributions (${\vec{\theta }}_{j}$).

Let *A*_*i *_be an indicator variable drawn from the location space ${\left\{0,1\right\}}^{\left(L_{i}-w+1\right)}$ of sequence *i*, **A **= [*A*_1_, ..., *A*_*i*_, ..., *A*_*N*_]^*T *^be the set of indicator variables representing the motif start sites (i.e. a local alignment) in the sequences, and *w *be the motif width. The total number of local alignments (*V*) can be generally expressed as: $V=\prod_{i=1}^{N}\left(\begin{array}{l} L_{i}-w+1\\ \left|A_{i}\right| \end{array}\right)$, and here the number of motif sites on sequence *i *is defined as: |*A*_*i*_| = Σ_*l *_*A*_*il*_. Therefore, if |*A*_*i*_| = 1 for all *i*, it is an *oops *model, otherwise it is a *zoops *or multiple-site model. The total number of motif sites is |**A**| = Σ_*i *_|*A*_*i*_|. Alternatively, an indicator variable *a*_*i *_= *l *is used to represent the motif starting at position *l *on sequence *i*, which is equivalent to *A*_*il *_= 1. Note that *a*_*i *_= 0 means no motifs found on sequence *i*. If multiple sites occur on a sequence, a vector (${\vec{a}}_{i}$) is used to store all the positions. Obviously a motif indicator vector ${\vec{a}}_{i}\in P_{i}$ ({1, 2, ..., *L*_*i *_- *w *+ 1}), here $P_{i}$ is the power set of the *i*-th sequence motif sites. The alignment of motif sites is initialized by randomly generating a set of motif start sites (i.e. **A**^(0) ^or equivalently ${\left[{\vec{a}}_{1}^{\left(0\right)},...,{\vec{a}}_{N}^{\left(0\right)}\right]}^{T}$) and then it is progressively refined until convergence.

### Evaluating the weight function

The information weight function defined in equation (1) needs estimation of two parameters: motif (${\vec{\theta }}_{j}$) and background (${\vec{\theta }}_{0}$). Suppose a counting matrix derived from a DNA motif alignment, {*c*_*jk*_}^*w *× 4^, here *c*_*jk *_is the number of the nucleotide *k *counted on position *j *in the motif alignment used, and then the information weight function (*τ*_*jk*_), as originally defined by Kullback and Leibler in 1951 [22], is computed as,

(11) $$\tau_{jk}=\log\frac{\theta_{jk}}{\theta_{0k}}=\log\frac{c_{jk}+\beta_{k}}{\sum_{k=1}^{\left|K\right|}c_{jk}+|\vec{\beta }|}-\log\theta_{0k},$$

where *τ*_*jk *_is the information weight of the base *k *on motif position *j*. The pseudo-counts $\vec{\beta }$ are added to avoid zero counting in a motif alignment due to small sample, *β*_*k *_is the pseudo-count of the base *k*, and |$\vec{\beta }$| = Σ_*k *_*β*_*k*_. It is often set as follows: *β*_*k *_= 1.25/4 for a DNA sequence [33]. The background distribution ${\vec{\theta }}_{0}$ can be estimated from the sequences or user-specified. Note that the expectation of the information weight defines the relative entropy function [34] or information content [24].

### Overall objective function

Suppose each alignment is thought of as a hidden state in the alignment space, the motif discovery problem can be formulated as finding the optimized alignment state (v*) among the entire alignment space. Index a state by $v\equiv {\left[{\vec{a}}_{1},...,{\vec{a}}_{i},...,{\vec{a}}_{N}\right]}^{T}=A^{\left(v\right)}$, and let the potential energy of state *v *be *H*^(*v*) ^= *H*(**S**, **A**) where **A **is the alignment corresponding to the state *v*. The energy may be related to an alignment score or the motif sequence specificity/binding energy [23]. Then at equilibrium the probability of state *v*, *p*(*v*), is calculated as,

(12) $$p(v)=\frac{1}{Z(\lambda )}\exp\{-\lambda H(S,A)\}$$

where the normalizing constant is defined as,

$$Z(\lambda )=\int_{P_{1}}\cdots \int_{P_{N}}e^{-\lambda H(S,A)}d{\vec{a}}_{1}\cdots d{\vec{a}}_{N}.$$

Therefore, the optimized alignment state (v*) is the one with the maximum probability (p*). If v* is found, then the parameter estimation (Θ*) is done. However, computing the partition function or normalized constant (i.e. *Z*) is commonly intractable, because the alignment problem has proven *NP*-complete [4]. Markov chain Monte Carlo methods are frequently applied to approximate this kind of hard problem. This paper defined the maximum log-likelihood as the potential energy function, see equation (3).

### Metropolis-Hastings sampler

Since it is hard to draw a sample directly according to equation (11), the Monte Carlo optimization methods such as the Metropolis-Hastings sampling (MHS) algorithm are often employed to iteratively draw a series of samples according to a proposed probability distribution such as the one in equation (5). Therefore, the sequence local alignment can be formulated as a Markov chain Monte Carlo (MCMC) optimization problem. Note that MCMC is a general framework for approximation methods in search of a large space characterized by complicated or unknown functions.

In solving the sequence local alignment problem, MHS generates samples (e.g. **A**) from a probability distribution *p*(·), and explores the local alignment space Ω(**S**) using a Markov chain. MCMC does not sample directly from *p*(·), but only requires that the density *p*(*v*) can be evaluated within a multiplicative constant *p*(*v*) = $\tilde{p}$(*v*)/*Z *and $\tilde{p}$(*v*) is the un-normalized target distribution. In equation (4), $\tilde{p}$ = exp{-*λH*(·)}. A Markov chain is a discrete-time stochastic process {**A**^(0)^, **A**^(1)^, ⋯} with property that the state **A**^(*t*)^given all previous values {**A**^(0)^, **A**^(1)^, ⋯ **A**^(*t*-1)^} only depends on **A**^(*t*-1)^: P(**A**^(*t*)^|**A**^(0)^, **A**^(1)^, ⋯ **A**^(*t*-1)^) = *P*(**A**^(*t*)^|**A**^(*t*-1)^). We call *P*(·|·) the transition matrix of the Markov chain. *P*(·|·) is stationary, that is to say, independent of time or step (*t*).

The MHS sampler starts with any alignment **A**^(0) ^by randomly initializing a seed. Notice there exists a one-to-one map: **A**^(*t*) ^→ Θ^(*t*)^. Then the MHS algorithm iterates the following two steps: (1) Propose a random perturbation of the current state (*t*), i.e., **A**^(*t*) ^→ **A**', where **A**^' ^can be viewed as generating from a proposal distribution *P*(**A**^(*t*) ^→ **A**') = *P*(**A**'|**A**^(*t*)^) by a series of independent random sampling. Then, one can toss a Bernoulli coin with probability of *α*_*H *_coming up heads: if heads show up, accepts the new move: **A**^(*t*+1) ^= **A**'; otherwise stay as it was: **A**^(*t*+1) ^= **A**^(*t*)^.

### Parallel MHS samplers

MCMC algorithms are often sequentially applied. A single chain is run for a long time until it converges to a stationary distribution. The states visited during the initial stage (i.e. burn-in phase) are usually thought to be unreliable and thus samples are discarded if reconstructing the density. However, this burn-in time should be short enough (i.e. well-mixing) to ensure the computational efficiency. The burn-in time is indeed depending on the initial points. A helpful strategy to select a good seed is simply running multiple independent MCMC chains, each of which starts with a different seed [18]. Suppose there are *R *parallel chains each of which is independent, and let the ensemble of alignments be $\vec{v}$ = (*v*_1_, ⋯, *v*_*R*_), then the Boltzmann distribution of the ensemble is given rise to,

(13) $$p(\vec{v})=\prod_{r=1}^{R}p(v_{r})=\frac{1}{Z^{R}}\exp\{-\sum_{r=1}^{R}\lambda H(v_{r})\}.$$

The above function treats the temperature (*T*) as constant and can be easily extended to a temperature variable (*T*_*r*_) attached to a MCMC chain [20]. Notice that there are no information exchange among these chains. The population-based MCMC (i.e. PMC) simulates samples according to a pooled proposal distribution, that is, *v*_*r *_comes from the $\bar{p}$(*v*_*r*_) defined in equation (7) rather than the *p*(*v*_*r*_) in equation (4) as information exchange occurs among chains.

### Performance evaluation

The nucleotide-level accuracy (*nla*) metric is defined to evaluate the motif-finding algorithm performance based on the known alignment (**O**) and predicted alignment (**A**). Let *o*_*i *_be the known motif position of sequence *i*, and *a*_*i *_be the predicted motif position on the same sequence. The function *nla *is defined as,

(14) $$nla(O,A)=\frac{1}{\left|A\right|}\sum_{i=1}^{\left|A\right|}\frac{\left|a_{i}\cap o_{i}\right|}{w},$$

where |**A**| is the number of motif sites, |*a*_*i *_∩ *o*_*i*_| is the size of overlapping block between the predicted and observed motifs from the sequence *i*. The predicted alignment may exactly match the observed (*nla *= 1.0) or have some phase shifts (0.0 <*nla *< 1.0) or completely misaligned (*nla *= 0.0).

### Motif-finders and their parameter settings

Both IMC and PMC versions run the same MHS motif-finding algorithms, and they were executed in the same parameter settings. The PMC population size was *R *= 20 as the default in all cases if not specified otherwise. The MEME algorithm (version 3.5.4) was also tested in the JASPAR data sets, and its parameters were set as its defaults except that MEME was notified of running the *oops *model, and the exact motif lengths were given as its input like other algorithms tested. The Projection algorithm [27] was specifically designed to deal with the planted (*l*, *d*)-motif discovery, and it requires the input of the *l *and *d*. The Projection program version 0.42 was tested with the same simulation data. Note that other algorithms tested do not require the *d *input. For example, to run the planted (10,2)-motif case, Projection executes the following command: "*findmotif -l 10 -d 2 -M 20 -s seqfile*". Projection can be downloaded from the website [35].

The Weeder algorithm [28] is one of the best motif predictors in mammalian promoters [29], however, its performance may be largely dependent on the nucleotide composition of real promoter regions. Several pre-calculated tables of word frequencies for various species (6-mers and 8-mers) come with the Weeder software. To cope with this requirement, new word frequency tables (6-mer and 8-mer words) were generated, for example, in the uniform case, each word was simply given frequency at 1. For other zero-order Markov backgrounds, first compute a w-word with minimum frequency: *f*_*min *_= (**θ**_0*k*'_)^*w*^, here *k*' is the nucleotide with the least probability and adjust the value to 1.0, and accordingly other words with the frequency *f*_*word *_are set to ⌊*f*_*word*_/*f*_*min *_+ 0.5⌋ (personal communication with Dr. Giulio Pavesi). The Weeder program version 1.31 was run with all the simulated data like this: "*weederlauncher.out seqfile bk large A*", here *bk *is the generated word frequency table. The Weeder user's manual can be found on the website [36].

### Experimental data sets tested

The first example used is the gold standard CRP data set [5,8,9], which consists of 18 experimentally determined binding sequences (*w *= 22 bp) each being embedded within a 105 bp promoter sequence. The motif width is set as 22 base pairs long according to the verified site length. The local alignment is performed on both forward and reverse DNA strands. The motif discovery problem becomes more difficulty as the sequence length gets longer. To test the length (*L*) effect, the 18 verified sites were planted into different data sets, each being different sequence length: i.e. *L *= 100, 200, 400, 800 and 16,000 bp long. The simulated background sequences were generated based on the zero-order Markov model (${\vec{\theta }}_{0}$ = [0.302, 0.183, 0.209, 0.306]^*T*^), which was estimated according to the original CRP data set.

Besides the length effect, other major factors such as the degree of motif conservation also have heavily impact on the performance. The data sets of the planted (*l*, *d*)-motif problem were generated as follows: given a fixed *l*-mer short motif (*l *= 10 to 19 bp), one generates 20 background sequences (*N *= 20) each with 600 bp long (*L *= 600), and each background sequence was randomly embedded with a variant of the consensus. A variant motif is a substring derived from the consensus with exactly *d*-position mutation (*d *= 2 to 6). The background distribution used in simulation is ${\vec{\theta }}_{0}$ = [0.3, 0.2, 0.2, 0.3]^*T*^. Each simulation was repeated 20 times and all simulated data sets were fed to the five algorithms tested. All computing jobs were submitted to the Apple Xserve cluster facility located in Children's Mercy Hospital's main campus. Notice that the same motif sets were also planted in two other backgrounds: [0.25, 0.25, 0.25, 0.25] and [0.2, 0.3, 0.3, 0.2], and similar conclusions could be drawn (data not shown.)

The JASPAR web server [37] provides experimentally verified binding sites data sets of eukaryotic transcription factors (TFs). However, the vast majority of these binding data were obtained by the SELEX method [24] that tested randomly generated double-strand oligonucleotides rather than genomic DNA sequences (personal communication with Dr. Boris Lenhard). Note that these verified motifs are highly conserved, each being short sequence (i.e. 25 bp on average). To challenge the *de novo *motif algorithms, these verified sites were planted into randomly simulated background sequences, each being 500 bp. A subset of the JASPAR binding sites was used to test the algorithms in the paper, that includes those TFs with more than 25 verified binding sequences and the motif width is at least 9 bp (i.e *w *≥ 9), as detailed in Table 2.

## Competing interests

The author declares that they have no competing interests.

## Authors' contributions

CB performed the work and wrote the manuscript.
